# Unilateral Fenestration of Internal Jugular Vein With a Radiological Clue: A Rare Case Report and Literature Review

**DOI:** 10.7759/cureus.39863

**Published:** 2023-06-02

**Authors:** Anbarasi Madoure, Lokesh Kumar Penubarthi, Akshat Kushwaha, Arun Alexander

**Affiliations:** 1 Otolaryngology, Jawaharlal Institute of Postgraduate Medical Education and Research, Puducherry, IND; 2 Otorhinolaryngology, Jawaharlal Institute of Postgraduate Medical Education and Research, Puducherry, IND

**Keywords:** spinal accessory nerve, otolaryngology, case report, fenestration, internal jugular vein

## Abstract

The intricacies of human anatomy continue to astound, as underscored by this unusual case of a 45-year-old female patient who presented to our esteemed otolaryngology department with T3N1MO squamous cell carcinoma of the lip. The preoperative diagnostic imaging of this patient divulged an enigmatic venous anomaly involving the internal jugular vein (IJV). Our team meticulously orchestrated a wide local excision of the primary tumor and modified radical neck dissection with Abbe Estlander flap reconstruction. Identification of the anomaly during the preoperative phase helped in meticulous planning and preparation. Thus, the surgical team was well-prepared for neck dissection and successfully navigated the rare IJV fenestration without incurring nerve or vascular injuries. This remarkable case accentuates the importance of maintaining a profound understanding of potential anatomical aberrations while performing intricate surgical procedures such as neck dissections. Heightened awareness can circumvent inadvertent damage to critical structures, ultimately safeguarding patient well-being. In this captivating report, we explain the preoperative suspicion, intraoperative identification, and subsequent outcome of a rare fenestration of the IJV encountered during a challenging neck dissection.

## Introduction

Neck dissection is one of the most standard surgeries performed in the head and neck region. During neck dissection, the internal jugular vein (IJV) forms an important structure and landmark for identifying other neurovascular structures like the spinal accessory nerve and carotid artery. Understanding anatomical variations and anomalies is crucial for oncological clearance and avoiding spinal accessory nerve damage. Here we report a fenestrated IJV that we identified in preoperative imaging and carefully preserved intraoperatively during neck dissection.

## Case presentation

**Figure 1:**
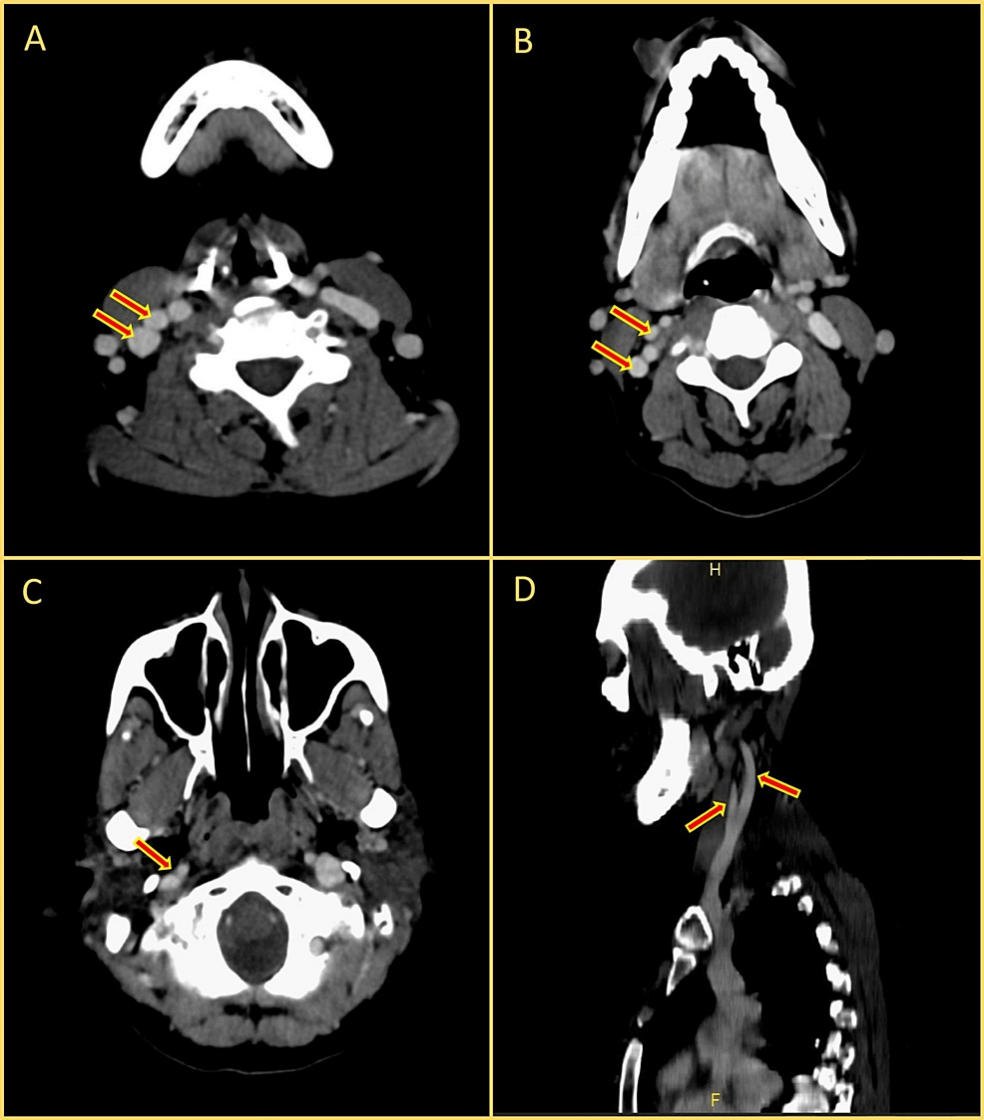
Preoperative contrast-enhanced computed tomography images showing the fenestration in axial and sagittal views A - Double IJV noted in axial view, B - Fenestration of IJV noted at the level of hyoid in axial view, C - Fusion of fenestrated IJV noted near exiting from jugular foramen in axial view, D- IJV fenestration in sagittal view. IJV: internal jugular vein

A 45-year-old female patient presented to our otolaryngology outpatient department with a painful growth over the right lower lip for four months. On examination, there was a 4x3cm ulceroproliferative growth involving the right lower lip with erythroplakia changes extending into the right buccal mucosa with a palpable right level 1B lymph node. Biopsy from the lesion showed well-differentiated squamous cell carcinoma. High-resolution ultrasonography of the neck showed a suspicious level 1B lymph node. Preoperative contrast-enhanced computed tomography (CT) imaging of the neck studied retrospectively revealed fenestration of IJV on the right side with normal left IJV (see Figure 1) suggesting the presence of IJV anomalies could be anticipated through preoperative imaging to avoid iatrogenic injuries during neck dissection. After tumour board discussion, the patient was taken for wide local excision of the oral commissural lesion with right modified radical neck dissection and Abbe Estlander flap reconstruction.

**Figure 2:**
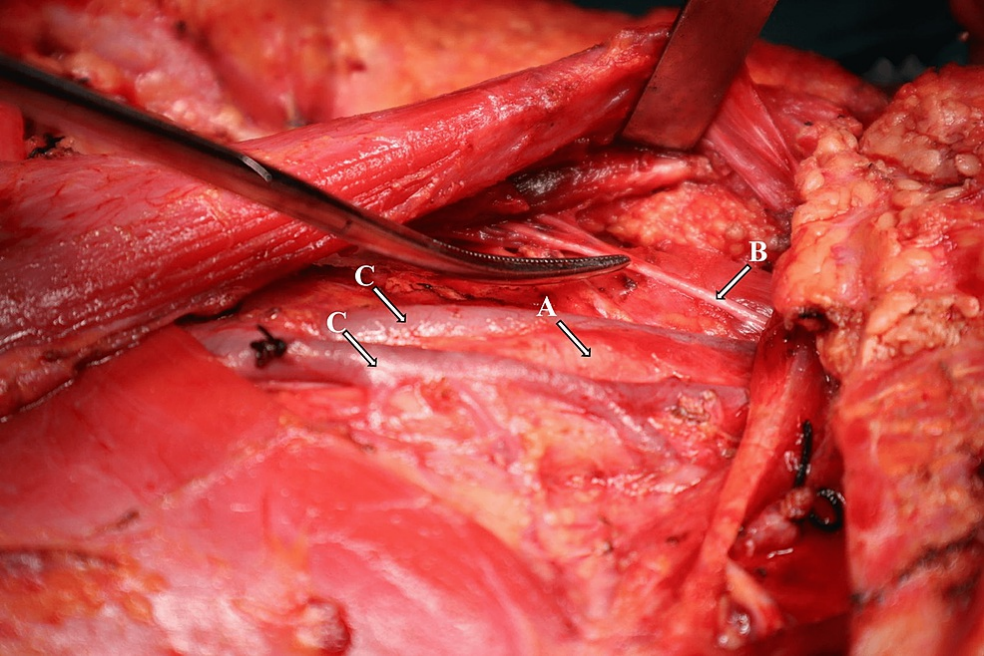
Intraoperative picture of the right neck dissection showing right IJV fenestration and its relation to spinal accessory nerve and common carotid artery Taken from the left shoulder of the patient showing retracted sternocleidomastoid muscle, fenestrated IJV, spinal accessory nerve, and common carotid artery. Arrow A shows the common carotid artery, arrow B shows the spinal accessory nerve, and the C arrows show the IJV fenestration. IJV: internal jugular vein

Intraoperatively, during neck dissection, a partial duplication of IJV, also known as fenestration, was observed just below the skull base till the level of the hyoid. The spinal accessory nerve passed posteriorly to IJV (see Figure 2), which was secured and preserved. Fibro-fatty tissue was dissected from all the levels of the neck. After achieving hemostasis, the wound was closed in layers. The postoperative period was uneventful, spinal accessory nerve function was intact, and the patient was discharged on postoperative day 5.

## Discussion

The IJV is the most prominent vein in the neck. The continuation of the sigmoid sinus forms it and exits via the jugular foramen, descends in the neck joins with the subclavian vein to form the brachiocephalic vein. Multiple craniocervical arterial fenestrations were described extensively, while venous fenestrations are rare in the cervical region [1]. Mumtaz et al. identified only 2% of IJV anomalies in a cohort of 1197 patients [2]. Most of the reported cases of IJV fenestration are found incidentally in the intraoperative period. Here we report a case of IJV fenestration which we witnessed in the preoperative imaging and counselled the patient about the possibility of spinal accessory nerve injury during neck dissection, which has not happened in our case. We discussed the incidence in our department's preoperative imaging findings, intraoperative details and implications of this rare IJV anomaly in patients undergoing surgery.

The prevalence of IJV fenestration ranges from 0.4% to 3.3% in multiple studies [3-5]. The prevalence of IJV fenestration in our institution is 0.5% (one in 500 cases from the year, 2015 to 2022). Very rarely, these patients present with neck swelling, dysphagia and dyspnea [6,7]; however, this patient had no pertinent symptoms to this anomaly. Though fenestration and duplication are used interchangeably, fenestration refers to the branching of a part of IJV and rejoining into a single vein before joining the subclavian vein [8]. In contrast, duplication refers to IJV becoming two veins and entering the subclavian separately. In our case, the IJV was split into two soon after exiting from the jugular foramen, travelled as two veins till the level of hyoid and reunited into one vein in the lower neck. In a review by Wang et al., the common carotid artery was lying behind the posterior division of the IJV as found intraoperatively [9]. Whereas, we found it lying in between the fenestration.

Towbin and Kanal [10] reported two cases of fenestrated IJV incidentally in CT angiography, predicting anomalous IJV before any procedure. Most documented case reports [3,9] were incidentally noted cases of IJV fenestration in the intraoperative period. Nevertheless, there will be a chance of injury to the IJV itself or the adjoining spinal accessory nerve if the IJV fenestration is encountered without prior knowledge. Preoperative or preprocedural knowledge of IJV duplications is essential not only to head neck surgeons but also to anaesthetists and physicians for placing a central venous line. In our case report, we suspected the possibility of IJV fenestration in the preoperative CT imaging. This helped us to counsel our patient for possible intraoperative spinal accessory nerve injury in this challenging case. We found the spinal accessory nerve behind the posterior division of IJV contrary to others [4]. The fibrofatty tissue around the fenestration and spinal accessory nerve was carefully dissected to preserve both structures. Postoperatively, the patient had no spinal accessory nerve paresis.

## Conclusions

Fenestration and duplication of the IJV are uncommon occurrences in clinical practice. Nevertheless, we conclude that it is essential for clinicians to be aware of this rare anatomical variation and consider it preoperatively. Thereby, the risk of accidental injury to the IJV and the spinal accessory nerve during surgery can be avoided, ensuring a safer and more successful operative outcome.
